# Comparison of the short-term and long-term effects of AngioJet and AcoStream mechanical catheter thrombolysis on acute iliofemoral vein thrombosis

**DOI:** 10.1016/j.clinsp.2026.101097

**Published:** 2026-09-18

**Authors:** Yaobo Yang, Xuan Dong, Yilin Zhao, Sipan Chen

**Affiliations:** Department of Intervention, Shaanxi Provincial People's Hospital, Xi'an, Shaanxi, China

**Keywords:** DVT, AngioJet, AcoStream, Catheter thrombolysis

## Abstract

**Objective:**

To compare the short-term and long-term clinical efficacy and safety of AngioJet and AcoStream thrombectomy devices in the treatment of acute iliofemoral venous thrombosis.

**Methods:**

We conducted a 4-year retrospective case ‒ control study. A total of 243 patients with acute iliofemoral deep vein thrombosis underwent endovascular interventional therapy in our hospital. According to different operation methods, patients were divided into two groups: Group A (AngioJet mechanical thrombectomy system) and Group B (AcoStream mechanical thrombectomy system). The clinical basic data and short-term postoperative efficacy of the two groups were compared. After 36-months of follow-up, the recurrence rate and long-term efficacy of post-thrombotic syndrome were compared between the two groups. The Kaplan-Meier survival method was used to analyze the proportion of Post-Thrombotic Syndrome (PTS).

**Results:**

The thrombus aspiration time in the AngioJet group (4.62 ± 0.12 min) was shorter than that in the AcoStream group (5.87 ± 0.09 min) (p = 0.001); the intraoperative blood loss (264.12 ± 5.46 mL) was less than that in the AcoStream group (307.62 ± 4.52 mL) (p < 0.001); the hemoglobin difference (15.57 ± 0.72 g/L) was less than that in the AcoStream group (24.58 ± 0.86 g/L) (p < 0.001); the D - D decrease rate (0.85 ± 0.03 mg/L/D) was faster than that in the AcoStream group (0.75 ± 0.03 mg/L/D) (p = 0.03); the dosage of urokinase (147.39 ± 0.71) million units was more than that in the AcoStream group (125.33 ± 1.01) million. Units (p < 0.001). The swelling reduction rates of the thigh and calf at 24-hours after surgery were (0.61 ± 0.01%) vs. (0.56 ± 0.01%) and (0.63 ± 0.01%) vs. (0.66 ± 0.01%), respectively, with statistically significant differences between the two groups (p < 0.05). The incidence of postoperative hematuria in the AngioJet group (91.9%) was higher than that in the AcoStream group (0.0%) (p < 0.001).

After 36-months of follow-up, the thrombus recurrence rate in the AngioJet group (8.9%) was compared with that in the AcoStream group (2.5%) (p < 0.05), and the incidence of postoperative PTS in the two groups was (34.95%) vs. (21.67%) (p = 0.016).

**Conclusion:**

Compared with the AcoStream system, the AngioJet system has a higher efficiency of thrombus clearance, but the incidence of hematuria and the recurrence rate are slightly higher. Of course, both have their advantages and disadvantages, and the appropriate surgical method should be selected according to the individual conditions of patients.

## Highlights

This study directly compares AngioJet and AcoStream mechanical thrombectomy devices to guide clinical selection.
It focuses on subacute lower limb DVT patients, filling the research gap in this population.
It evaluates short- and long-term outcomes comprehensively, reflecting real clinical benefits.

## Introduction

Acute venous thrombosis of lower extremities (e.g., Deep Vein Thrombosis, DVT) is a common clinical vascular emergency that can lead to serious complications such as pulmonary embolism and post-thrombotic syndrome, threatening patients' lives and long-term quality of life^1^ Of all hospital deaths, 7% are caused by acute PE^2^ Approximately 70% to 80% of PE cases are caused by acute DVT. In addition, 20% to 50% of patients with acute symptomatic proximal DVT patients subsequently develop PTS. Thrombolytic therapy, including Catheter-Based Thrombolysis (CDT)^3^ and Pharmacomechanical Thrombolysis and Thrombectomy (PCDT),^4^ has come to dominate clinical practice in recent decades for the treatment of acute proximal DVT. For patients with severe symptoms and low bleeding risk due to acute iliofemoral vein thrombosis, CDT (Catheter-directed thrombectomy) represents an important and effective vascular recanalization strategy that can rapidly alleviate symptoms and potentially reduce the severity of post-thrombotic syndrome^5^, ^6^ PCDT has been shown to be a more effective and safe treatment^7^

Multicenter follow-up results were published in the PEARL study, showing that 93% of the Class II to III thrombectomy cases were cleared for 329 AngioJet (Boston Scientific) thrombectomy cases.

AngioJet and Acostream, as two representative thrombectomy systems, have been widely used in clinical practice due to their high efficiency and minimally invasive characteristics^8^ However, the differences in mechanism of action, indications, and efficacy between the two remain to be further explored. AngioJet adopts the principle of fluid dynamics, generates negative pressure to suction thrombus through high-speed saline jet, and has the function of drug-mechanical synergy thrombolysis (PMT). Acostream, as a new rotating thrombectomy device, achieves thrombus fragmentation and aspiration through the mechanical rotation of a double-helical catheter, theoretically causing less damage to the vascular intima.

This article aims to review the principle of action, technical characteristics and clinical apExplore the application progress of Acostream and Angiojet, and compare and analyze the advantages and limitations of Acostream and Angiojet based on existing evidence, so as to provide a reference for the individualized treatment strategy of acute venous thrombosis of the lower limbs, and look forward to the direction of technical optimization in the future.

### Patient selection and study design

We conducted a 4-year retrospective case-control study. A total of 243 patients with acute iliofemoral deep vein thrombosis underwent endovascular interventional therapy in our hospital. According to different operation methods, the patients were divided into two groups: Group A (AngioJet mechanical thrombectomy system, 123 cases) and Group B (AcoStream mechanical thrombectomy system, 120 cases). Both groups received catheter ‒ directed thrombolysis after thrombectomy. The clinical data and postoperative efficacy were compared between the two groups. Postoperative follow-up was conducted for 36-months. Then, the recurrence rate and Post-Thrombotic Syndrome (PTS) in the two groups were compared, and the Kaplan-Meier survival method was used to analyze the proportion of Post-Thrombotic Syndrome (PTS). This study was approved by the Ethics Committee of Shaanxi Province People's Hospital, and all patients signed the informed consent form.

### Inclusion criteria

(1) DVT confirmed by computed tomography venography (CTV) and serial Doppler ultrasound meeting diagnostic criteria related to LDVT in the Guidelines for the Diagnosis and Treatment of Deep Vein Thrombosis.^9^ (2) age < 80 years; (3) involvement of iliac and common femoral veins with or without involvement of other veins; (4) no history of DVT or PE.

### Exclusion criteria

(1) severe contraindications to anticoagulation and/or thrombolysis, including active bleeding, history of severe trauma or major surgery within the first 4 weeks, gastrointestinal bleeding or cerebral hemorrhage within the first 3 months, uncontrolled hypertension (systolic blood pressure > 180 mmHg or diastolic blood pressure > 110 mmHg)^10^, ^11^, ^12^ (2) severe renal insufficiency (creatinine clearance [CrCl], < 30 mL/min) or allergy to contrast media; and/or; (3) thrombosis involving the inferior vena cava.

### Surgical treatment

Both groups were placed in the supine position and given local anesthesia. The common femoral vein on the unaffected side was selected as the access site, and an inferior vena cava filter was inserted. The patients were then turned to the prone position, and the perforation of the popliteal vein on the affected side was performed under ultrasound guidance. In the AngioJet group, a 6F sheath was inserted, while in the AcoStream group, a 12F sheath was used. Low molecular weight heparin (70–100 U/kg) was administered, followed by thrombectomy.

**Fig. 1:**
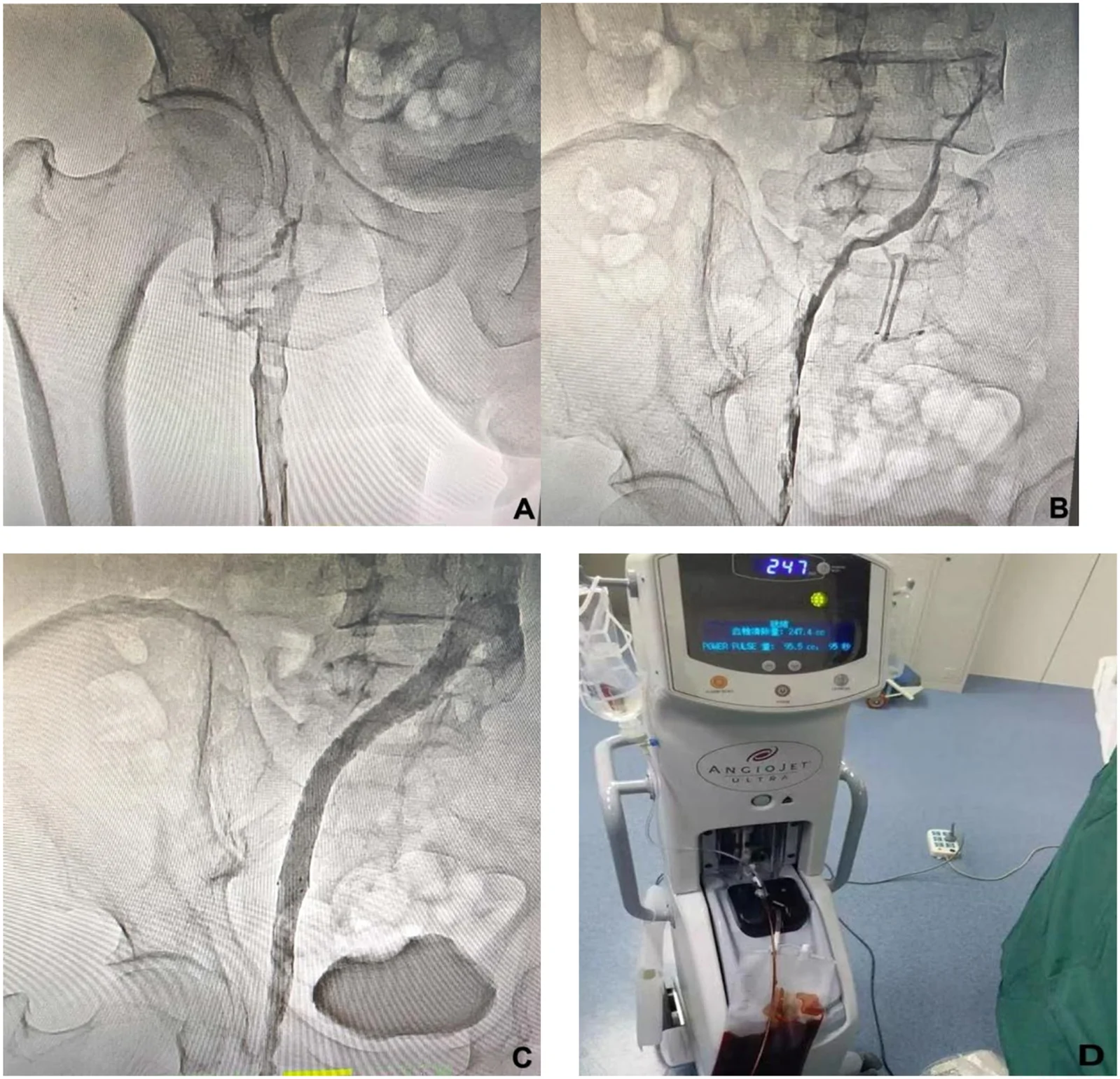
AngioJet thrombus aspiration for right AIFVT. (A) Supine position. Angiography shows acute thrombosis of the right iliac-femoral vein; (B) Common iliac vein stenosis after AngioJet thrombectomy; (C) Patency of blood flow after stent implantation; (D) AngioJet machine and aspiration of thrombus.

AngioJet group: With the guidance of the guidewire, the 6F AngioJet catheter manufactured by Boston Scientific was inserted into the lesion segment. The machine was first adjusted to the spray mode to administer 200,000 to 500,000 units of urokinase for thrombolysis within the lesion segment, lasting approximately 30-minutes. Subsequently, the mode was switched to aspiration to perform thrombus aspiration, advancing slowly toward the proximal end at a speed of 1 to 3 mm/s. After completion of aspiration, a DSA was performed. If residual thrombus segments were present, aspiration was repeated 1- to 2-times as needed, with the total aspiration time controlled within 480 seconds. During aspiration, close monitoring of the patient's heart rate and blood pressure changes was required, along with observation for symptoms such as palpitations and chest tightness (Fig. 1).

**Fig. 2:**
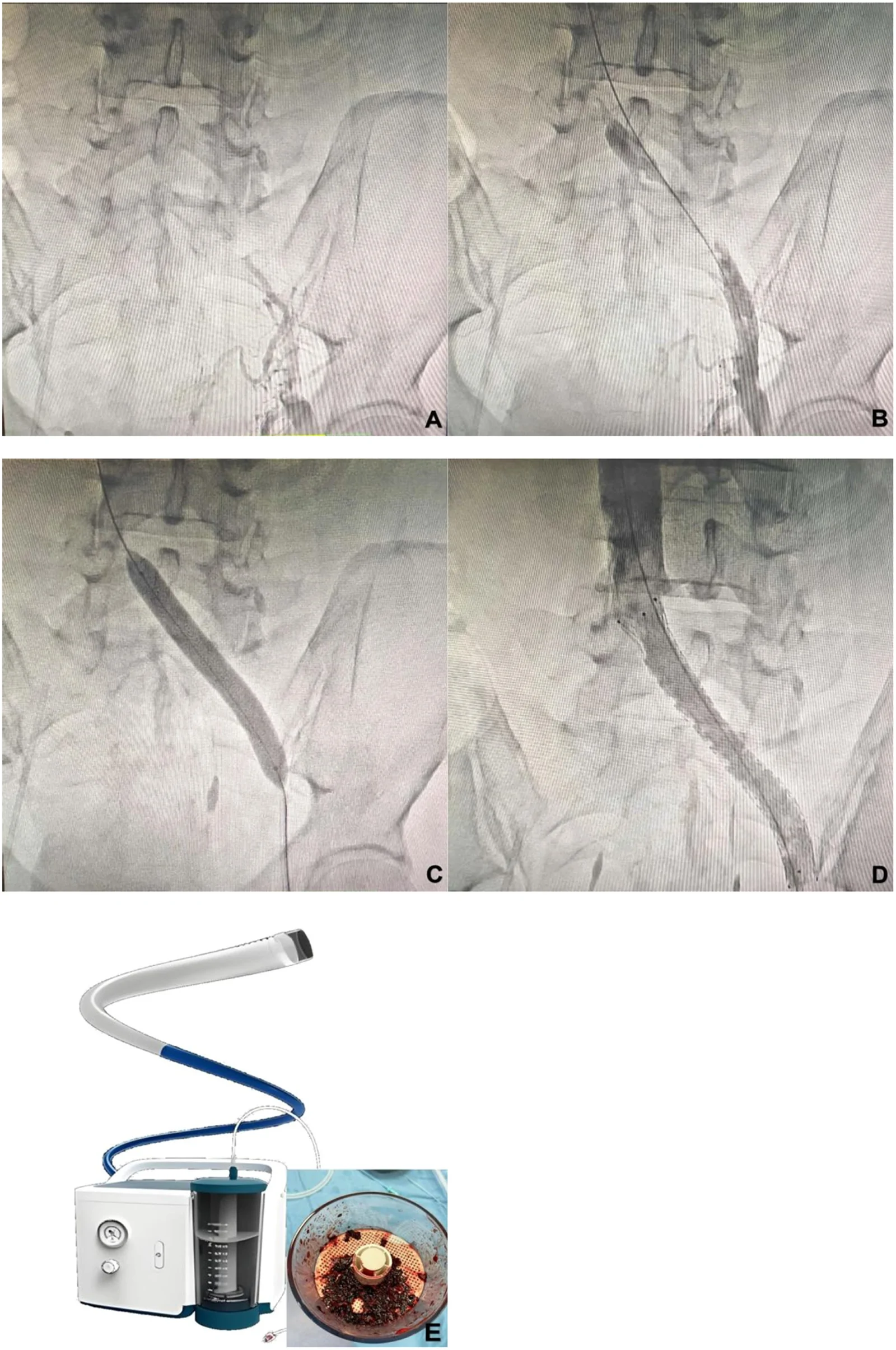
AcoStream thrombus aspiration in left AIFVT. (A) Supine position. Angiography shows acute thrombosis of the left iliac-femoral vein; (B) Common iliac vein stenosis after AcoStream thrombectomy; (C) Patency of blood flow after stent implantation; (D) AcoStream machine and aspiration of thrombus.

AcoStream group: Under the guidance of the guidewire, the 12F AcoStream catheter manufactured by Beijing Xianruida Medical Technology Co., Ltd. was inserted. The catheter was connected to a negative pressure cylinder via a connecting tube, with the volume-marked negative pressure cylinder mounted on a negative pressure pump set at 1 nominal atmosphere (101.325 kPa). The procedure involved repeated segmental suctioning from the proximal to the distal end of the thrombus: if rapid blood flow was detected during retraction, the procedure was paused and the catheter was retracted for re-suction; if slow blood flow was observed, the connecting tube could be disconnected, or 10 mL of heparinized saline solution (2000 IU heparin added to 300 mL saline) could be aspirated. After each suction cycle, a DSA re-examination was performed. If continuous blood flow was not restored and residual thrombus was detected, suctioning was repeated until the DSA indicated thrombus clearance (Fig. 2).

If the thrombectomy in both groups is satisfactory but the degree of iliac vein occlusion or compression exceeds 85%, primary iliac vein stenting is performed^13^ After thrombectomy, CDT-assisted therapy is administered: thrombolytic catheter is connected via a micro-pump, and urokinase (10 × 10^4 U/0.5 h) is continuously administered in a sequential pulse manner over 24-hours. During thrombolysis, prothrombin time, activated partial thromboplastin time, and plasma fibrinogen concentration are monitored every 12-hours, while D-dimer and platelet count are monitored every 4-hours. When fibrinogen concentration reaches 1.5 g/mL, the urokinase dose is halved; when fibrinogen concentration drops to 1.0 g/L, heparinized saline is used as an alternative. If clinical bleeding evidence such as hematuria occurs, fresh frozen plasma and cryoprecipitate are administered intravenously. After 3-days of continuous treatment, angiography is performed for re-evaluation, and low molecular weight heparin (5000 U) is subcutaneously injected every 12-hours daily.

### Assessment and follow-up: the primary endpoints were short-term thrombolytic efficacy and safety

Postoperative assessment of lower limb swelling reduction was conducted 24-hours after surgery, with recording of the difference in lower limb diameter before and after treatment, swelling reduction rate, catheter retention time, urokinase dosage, d-dimer decline rate, hospitalization duration, and complication incidence. According to the recommendations of Porter et al.,^11^ two independent evaluators scored venous patency through venography: the lower limb veins were divided into 7-segments, including the inferior vena cava, common iliac vein, external iliac vein, common femoral vein, proximal femoral vein, distal femoral vein, and popliteal vein. The scoring criteria were: 0-points for complete patency, 1-point for partial patency, and 2-points for complete occlusion. The venous patency rate was calculated as: (patency score before thrombolysis-patency score after thrombolysis)/ patency score before thrombolysis × 100%. The thrombus clearance rate grading reference criteria^12^^,^^13^ were: Grade III indicated thrombus clearance > 90%, Grade II indicated 50%∼90%, and Grade I indicated < 50%. The degree of swelling reduction in the affected limb was assessed by the preoperative and postoperative thigh circumferential difference (difference in circumferential measurement at 15 cm above the patellar margin between the affected and unaffected sides) and the calf circumferential difference (difference in circumferential measurement at 10 cm below the patellar margin between the affected and unaffected sides). The swelling reduction rate in the affected limb was calculated as: (preoperative circumferential difference of the affected limb-postoperative 24-hour circumferential difference of the affected limb) / preoperative circumferential difference of the affected limb × 100%^14^

After discharge from the hospital, it is routine to take rivaroxaban tablets (Bayer) orally for 3-weeks (15 mg, twice daily) + 3-months (20 mg, once daily) + 3-months (10 mg, once daily). For those with iliac vein stent implantation, continue to take aspirin enteric-coated tablets (Bayer) 100 mg orally, once daily, for 2- to 3-years. Wear elastic stockings for 1- to 3-months. The incidence rates of PTS at 1, 3, 6, 12, 24, and 36-months after discharge were the secondary study endpoints. All patients were followed up in the outpatient clinic, and assessments were performed using Doppler ultrasound combined with CTV. Venous patency and Villalta scores were evaluated based on the results and complications^15^^,^^16^ The Villalta score includes^17^ patient symptoms (pain, skin itching, heaviness, paresthesia, and spasms) and signs (skin induration, pretibial edema, pigmentation, skin redness, superficial venous dilation, and gastrocnemius tenderness) score. Higher scores indicate increasing severity^18^ Patients are then classified as having no PTS (score, 0‒4), mild PTS (score, 5‒9), moderate PTS (score, 10‒14), or severe PTS (score 15 or the presence of ulcers).

### Statistical analysis

Data were analyzed using SPSS 27.0 software. Measurement data were expressed as (x±s). The two-independent-samples *t*-test was used for comparison between groups. Count data were expressed as n (%). The χ^2^ test was used for comparison between groups. The Kaplan-Meier survival method and log-rank test were used to analyze the proportion of Post-Thrombotic Syndrome (PTS); p < 0.05 was considered statistically significant.

## Result

This study retrospectively analyzed a total of 243 patients, with no deaths or severe adverse events such as major cardiovascular or cerebrovascular accidents occurring during follow-up. The mean age of patients was 69.76±8.9 years (range: 40–79 years), including 122 males and 121 females. No statistically significant differences were observed in baseline characteristics between the two groups (p > 0.05).

The efficacy of the two mechanical thrombectomy systems in terms of thrombectomy rate levels was comparable, with no statistically significant differences (p > 0.05) (Table 1).

**Table 1:** Comparison of baseline characteristics between the two groups [n (%)].

	AngioJet group (n = 123)	Acostream group (n = 120)	χ^2^	p-value
**Male**	**67 (54.5)**	**55 (45.8)**	**1.81**	**0.18**
**Thrombus range**				
Iliac vein	27 (21.9)	30 (25.0)	1.71	0.42
Iliofemoral DVT	51 (41.4)	40 (33.3)		
Full-limb mixed DVT (iliac vein to calf vein)	45 (36.6)	50 (41.7)		
**Hypertension**	72 (58.5)	6 0 (50.0)	1.78	0.18
**Postoperative hypertension increase**	102 (82.9)	5 (4.2)	152.89	<0.001
**Thrombotic cause (%)**				
Brake	67 (54.5)	55 (45.8)	2.73	0.60
Bedridden	21 (17.0)	30(25.0)		
Trauma	20 (16.3)	20 (16.7)		
Thrombophilia	10 (8.1)	10(8.3)		
Unknown aetiology	5 (4.1)	5 (4.2)		
**Thrombolysis rate**				
≦50%	5 (4.1)	5 (4.2)	5.01	0.08
50%‒90%	30 (24.4)	45 (37.5)		
≧90%	88 (71.5)	70 (58.3)		
**Acute recurrence**	11 (8.9)	3 (2.5)	4.64	0.031
**Hemoglobinuria**	113 (91.9)	0 (0.0)	206.07	<0.001
**Iliac vein stent placement (%)**	36 (30.0)	35 (29.2)	0.00	0.98

DVT, deep vein thrombosis.

The thrombus aspiration time in the AngioJet group (4.62 ± 0.12 min) was shorter than that in the AcoStream group (5.87 ± 0.09 min) (p = 0.001). The intraoperative blood loss (264.12 ± 5.46 mL) was less than that in the AcoStream group (307.62 ± 4.52 mL) (p < 0.001). The hemoglobin difference (15.57 ± 0.72 g/L) was less than that in the AcoStream group (24.58 ± 0.86 g/L) (p < 0.001). The D - D decrease rate (0.85 ± 0.03 mg/L/D) was faster than that in the AcoStream group (0.75 ± 0.03 mg/L/D) (p = 0.03). The dosage of urokinase (147.39 ± 0.71 million Units) was more than that in the AcoStream group (125.33 ± 1.01 million Units) (p < 0.001) (Table 2).

**Table 2:** Comparison of curative effect between the two groups before and after operation [(x±s)].

	AngioJet group (n = 123)	Acostream group (n = 120)	*t*	p-value
**Age, years**	70.63±0.82	68.87±0.79	−1.53	0.12
**Symptom duration, hours**	2.92±0.14	2.87±0.13	−0.26	0.79
**Thrombus aspiration time, minute**	4.62±0.12	5.87±0.09	7.80	0.001
**Intraoperative blood loss, mL**	264.12±5.46	307.62±4.52	6.11	<0.001
**Hospitalization, days**	6.12±0.09	6.04±0.10	−0.56	0.57
**Diameter difference before treatment, cm**				
Above knee 15 cm	6.95±0.08	7.17±0.12	1.44	0.14
Below knee 15 cm	5.62±0.08	5.68±0.08	0.50	0.61
**Diameter difference after treatment, cm**				
Above knee 15 cm	2.63±0.04	3.04±0.06	5.06	< 0.001
Below knee 15 cm	1.97±0.05	1.85±0.04	−1.83	0.06
**Detumescence rate at 24-hours postoperatively, %**				
Above knee 15 cm	0.61±0.01	0.56±0.01	−3.44	0.001
Below knee 15 cm	0.63±0.01	0.66±0.01	2.07	0.04
Preoperative hemoglobin, g/L	134.01±0.84	134.66±0.91	0.52	0.60
Postoperative hemoglobin, g/L	118.43±0.89	110.08±0.73	−7.17	< 0.001
hemoglobin difference, g/L	15.57±0.72	24.58±0.86	7.99	< 0.001
*Preoperative PT, sec*	11.61±0.10	10.91±0.22	−2.95	0.004
*Postoperative PT, sec*	12.29±0.10	12.45±0.09	1.13	0.25
Preoperative Fg, g/L	2.53±0.05	2.47±0.05	−0.84	0.39
Postoperative Fg, g/L	2.98±0.04	3.00±0.05	0.32	0.74
Preoperative d-dimer, mg/L	4.81±0.08	4.59±0.08	1.81	0.07
Postoperative d-dimer, mg/L	2.19±0.07	2.38±0.06	−2.06	0.04
Descent velocity (mg/L/D)	0.85±0.03	0.75±0.03	2.08	0.03
Dosage of urokinase, million units	147.39±0.71	125.33±1.01	−17.93	< 0.001
Catheter retention time, hours	3.13±0.06	3.20±0.05	0.84	0.39

The swelling reduction rates of the thigh and calf at 24 h after surgery were (0.61 ± 0.01%) vs (0.56 ± 0.01%) and (0.63 ± 0.01%) vs (0.66 ± 0.01%), respectively, with statistically significant differences between the two groups (p < 0.05). The incidence of postoperative hematuria in the AngioJet group (91.9%) was higher than that in the AcoStream group (0.0%) (p < 0.001).

**Fig. 3:**
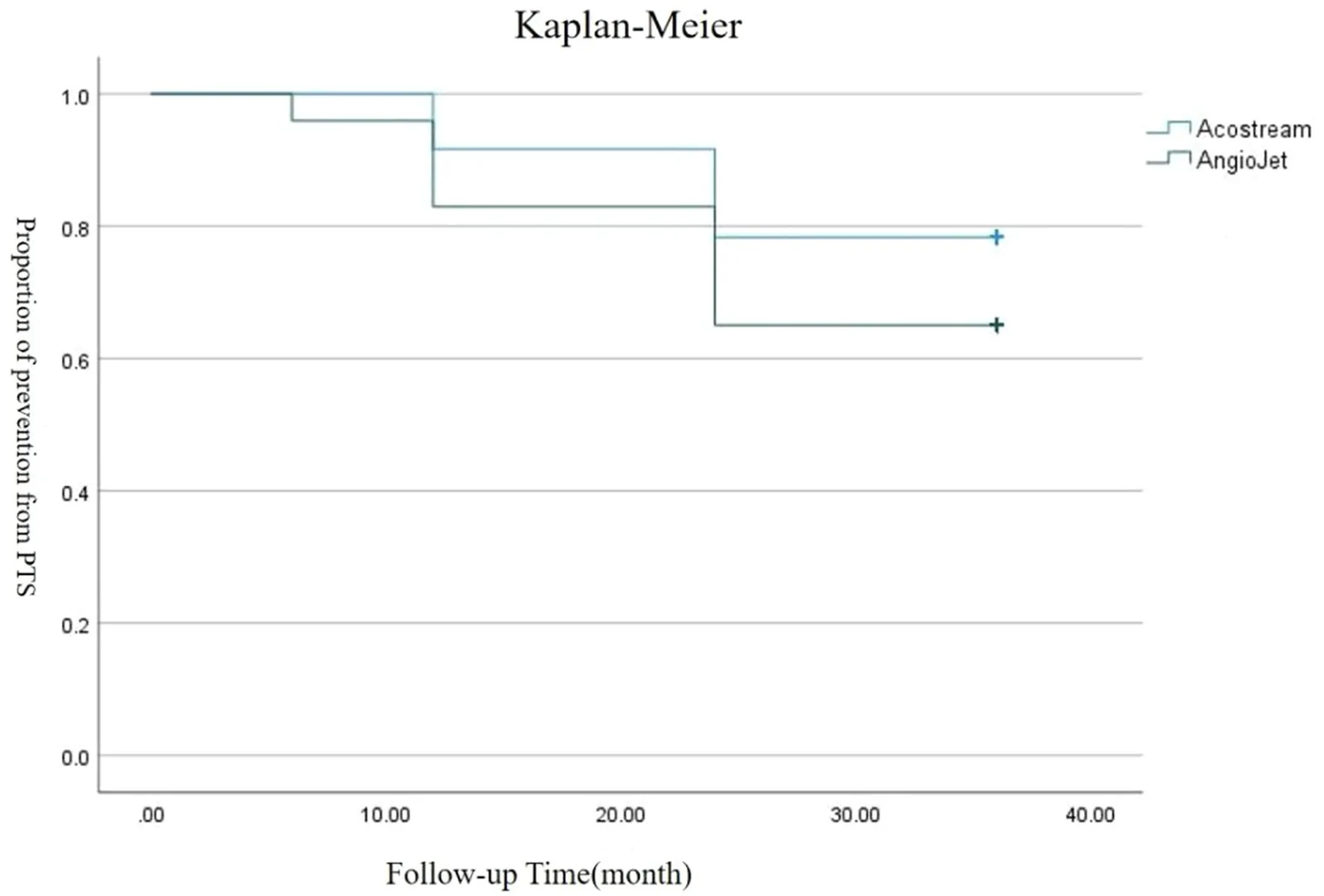
Kaplan-Meier Curve Analysis of Post-Thrombosis Syndrome (PTS) Stratified by treatment group the PTS incidence at 24-months after surgery was 34.95% in the AngioJet group and 21.67% in the AcoStream group (p = 0.016).

After 36 months of follow - up, the thrombus recurrence rate in the AngioJet group (8.9%) vs. that in the AcoStream group (2.5%) (p < 0.05), and the incidence of postoperative PTS in the two groups was (34.95%) vs. (21.67%) (p = 0.016) (Table 3, Fig. 3).

**Table 3:** Villalta score of postoperative Follow-up in the two groups.

Time (months)	Group	0‒4	5‒9	10‒14	≥15	p-value
3	AngioJet	123	‒	‒	‒	0.016
	Acostream	120	‒	‒	‒	
6	AngioJet	118	5	‒	‒	
	Acostream	120	‒	‒	‒	
12	AngioJet	102	16	‒	‒	
	Acostream	110	10	‒	‒	
24	AngioJet	80	17	5	‒	
	Acostream	94	12	4	‒

## Discussion

Acute iliofemoral deep vein thrombosis (DVT) is a common emergency in interventional vascular surgery. Inappropriate management may lead to severe complications such as pulmonary embolism and post-thrombotic syndrome (PTS),^19^ which significantly impair patients' quality of life. Conventional anticoagulation can only prevent thrombus propagation but fails to achieve rapid thrombus removal. Although catheter-directed thrombolysis (CDT) is more targeted than systemic thrombolysis, it is limited by prolonged lysis time and high bleeding risk^20^ In recent years, percutaneous mechanical thrombectomy (PMT) has developed rapidly,^21^ represented by the AngioJet and AcoStream systems. With the advantages of rapid thrombectomy and reduced thrombolytic agent dosage, these systems have become important therapeutic modalities for acute lower extremity DVT, especially iliofemoral DVT^22^, ^23^ This study compared the short- and long-term efficacy of AngioJet and AcoStream in the treatment of acute iliofemoral DVT to provide evidence for clinical decision-making.

The AngioJet mechanical thrombectomy device has a long clinical history. Based on the Bernoulli principle, it generates local negative pressure via high-velocity fluid jets to fragment and aspirate thrombi. This feature confers distinct advantages in emergency scenarios requiring rapid revascularization (e.g., phlegmasia cerulea dolens) and in patients with large thrombus burden^24^ AcoStream is a new-generation peripheral thrombectomy system consisting of a vacuum aspiration pump, disposable collection chamber, connecting tubing, and aspiration catheter set. Compared with manual aspiration using conventional large-bore catheters, AcoJet delivers stronger and more consistent negative pressure and offers a full range of catheter sizes to accommodate various vessel lumens. Its soft atraumatic tip minimizes intimal injury, while the rigid proximal shaft provides robust support. The filtration module in the collection chamber separates thrombus from blood, facilitating estimation of intraoperative blood loss^25^, ^26^ Additional benefits include sustained vacuum aspiration, reduced distal embolization risk, and low dependency on thrombolytic drugs^27^ The present study demonstrated that both systems achieved favorable technical success and immediate thrombus clearance rates with no statistically significant intergroup difference. However, the AngioJet group had significantly shorter thrombectomy time and less intraoperative blood loss than the AcoStream group, indicating higher efficiency and minimally invasive performance, likely attributable to its larger aspiration force and contact area^28^ AcoStream relies on a vacuum bottle for aspiration, which features a smaller aspiration area, longer procedure time, and greater adjacent tissue damage, resulting in higher blood loss. Meanwhile, total urokinase dosage was higher in the AngioJet group, as the system directly injects thrombolytics into the thrombus and amplifies drug distribution via intraluminal jet-induced negative pressure, thereby accelerating thrombolysis. In contrast, the aspiration mechanism of AcoStream partially replaces pharmacologic effects, reducing urokinase consumption. This is similar to the results of^29^ Tao Kang's comparison of the efficacy between catheter - directed thrombolysis and AngioJet pharmacomechanical catheter - directed thrombolysis in the treatment of acute iliofemoral deep vein thrombosis. However, his control group was catheter - directed thrombolysis, and the risk of bleeding with simple catheter thrombolysis was significantly increased.

Notably, the incidence of postoperative hematuria was markedly higher in the AngioJet group than in the AcoStream group (91.9%vs 0.0%). This is attributed to mechanical destruction of erythrocytes by high-pressure jets, causing transient hemoglobinuria that typically resolves spontaneously within 24–48 h postoperatively without long-term renal impairment. Nevertheless, close monitoring is essential. Intraoperative aspiration time should be precisely controlled, and perioperative hydration and urinary alkalization should be administered to reduce hemoglobinuria risk and protect renal function. AcoStream employs a 12F large-bore catheter with manual vacuum aspiration, causing minimal erythrocyte damage and extremely low hematuria rates, making it safer for patients with renal insufficiency or advanced age. The incidences of other complications, including vascular injury and puncture-site hematoma, were comparable between groups, and no procedure-related deaths occurred, confirming the high safety profile of both techniques.

Wu et al^30^ and Chen et al^31^ reported similar effects of the two mechanical thrombectomy systems on improving the differences in thigh/calf circumference and coagulation parameters. However, the present study found that the AngioJet group showed a faster decline in D-dimer levels at 24 h postoperatively and a higher thigh swelling resolution rate, but a lower calf swelling resolution rate than the AcoStream group. Large vessels such as the iliofemoral vein represent the main thrombotic burden, with wide lumens, abundant blood flow, and mostly fresh, soft thrombi. The powerful aspiration of AngioJet excels at clearing fresh thrombi in these major vessels and rapidly restoring patency, leading to prompt relief of venous outflow obstruction and marked thigh detumescence. During thrombectomy, AngioJet releases abundant thrombus fragments into the circulation, which are recognized as fibrin to be degraded, strongly activating the endogenous fibrinolytic system. This causes an initial sharp increase in D-dimer followed by rapid decline, reflecting the efficiency of fragment clearance and indirectly confirming extensive early thrombus removal. Despite multiple catheter options, AngioJet catheters are less flexible than slender thrombectomy catheters and may not fully reach or traverse highly tortuous distal calf deep veins. Calf veins are small and thin-walled; the strong negative pressure of AngioJet tends to appose the vessel wall, increasing the risk of endothelial injury, spasm, or even perforation. Operators therefore exercise greater caution in distal small vessels, reducing aspiration intensity and frequency and compromising thrombectomy efficacy. Furthermore, lower extremity DVT is often ascending, originating in the calf and extending proximally. Calf thrombi are therefore frequently older, organized, and tightly adherent to the vessel wall. AngioJet’s rheolytic aspiration performs optimally for fresh, soft clots but is less effective for organized, adherent thrombi. In comparison, AcoStream is less aggressive and carries lower endothelial injury risk in distal small vessels, allowing surgeons to operate more confidently in calf veins. Designed to capture and remove thrombi rather than merely fragment and disperse them, AcoStream reduces excessive fibrinolytic activation and achieves more complete removal of solid, distal thrombi resistant to natural fibrinolysis, resulting in lower residual thrombus burden and more thorough calf detumescence.

Multiple randomized controlled trials have documented the benefits of pharmacomechanical catheter-directed thrombolysis (PCDT) for venous patency and PTS prevention^32^ However, the largest trial, the ATTRACT trial, contradicted earlier findings by showing no PTS prevention with PCDT. Among 92 DVT patients with symptom duration ≤ 14 days, PTS rates at 2-year follow-up were similar between the PCDT and anticoagulation-alone groups. A subgroup analysis of 300 femoropopliteal DVT patients in ATTRACT also revealed no differences in PTS incidence or severity^33^ In contrast, a subgroup of 391 iliofemoral DVT patients in ATTRACT showed reduced moderate-to-severe PTS incidence and severity in the PCDT group, suggesting that patients with acute iliofemoral DVT are more likely to benefit from PCDT. PTS risk is directly correlated with DVT symptom onset and thrombus freshness^34^ Accordingly, American Heart Association guidelines recommend CDT or PCDT for patients with acute iliofemoral DVT (symptom duration < 21 days) and low bleeding risk^35^ The ATTRACT trial enrolled patients with symptom duration ≤ 14 days. Similarly, Mewissen et al^36^ reported grade Ⅲ complete thrombolysis in 34% of acute DVT patients (symptom duration ≤ 10 days) versus 19% of chronic DVT patients (symptom duration ≥ 10 days).

At 6–36 months of follow-up, the AngioJet group exhibited higher thrombus recurrence rate, PTS incidence, and Villalta score than the AcoStream group. Three key mechanisms underlie these findings: Mechanical manipulation by AngioJet causes more extensive intimal injury. Exposed subendothelial collagen is a potent thrombogenic stimulus that immediately activates platelets and the coagulation cascade, creating a nidus for thrombus recurrence. AngioJet achieves high clearance of acute fresh thrombi but may cause severe damage and leave residual thrombi when forcibly aspirating old, wall-adherent clots. AcoStream causes mild intimal injury and preserves an intact smooth intimal surface, reducing thrombogenicity. Additionally, ultrasound-assisted lysis combined with low-dose thrombolytics more effectively treats organized thrombi, leaving minimal residues and lowering recurrence risk^37^ Severe intimal damage and hemolysis induced by AngioJet trigger stronger local and systemic inflammatory responses, which further promote thrombosis^38^

The deep veins of the lower extremity contain numerous delicate valves. High-velocity water jets and aspiration from AngioJet readily cause irreversible mechanical damage to these fragile valves, leading to valvular insufficiency — the core pathological basis of PTS. AcoStream’s ultrasound energy and low-dose drugs have little impact on valve architecture, thus better preserving valvular function. Severe intimal injury during healing also induces excessive venous wall fibrosis, resulting in stiffness, reduced elasticity, and luminal stenosis, which further impede blood flow and exacerbate PTS. AcoStream’s milder injury leads to attenuated postprocedural fibroproliferation. More severe valvular and venous wall damage in the AngioJet group translates to more severe clinical manifestations (pain, swelling, skin pigmentation, ulceration), reflected in higher Villalta scores^39^ Such underlying damage predisposes patients to earlier PTS onset and more rapid progression.

This study has several limitations. Long-term medication adherence and follow-up management remain challenging in China, similar to other countries; addressing these issues requires collaborative efforts in health education and management among governments, hospitals, and the public. Furthermore, this study does not provide new insights into controversial issues such as optimal DVT symptom duration, thrombolytic agent selection and dosage, or thrombectomy device choice. Nevertheless, it reflects real-world clinical practice in primary-level hospitals.

## Conclusion

Compared with the AcoStream system, the AngioJet system has a higher efficiency of thrombus clearance, but the incidence of hematuria and the recurrence rate are slightly higher. Of course, both have their advantages and disadvantages, and the appropriate surgical method should be selected according to the individual conditions of patients.

## Ethics approval and consent to participate

This study is a retrospective case-control observational study. All clinical data were retrospectively collected from previous medical records, and no additional invasive interventions, experimental treatments, or exposure treatments were administered to the patients. The data were anonymized and de-identified to protect patient privacy. Therefore, this retrospective analysis did not require approval from an ethics committee or a study protocol number. All methods were performed in accordance with the relevant guidelines and regulations.

## Data availability

The datasets generated and analysed during the current study are not publicly available due to a further study of this area but are available from the corresponding author on reasonable request.

## CRediT authorship contribution statement

**Yaobo Yang:** Data curation, Writing – original draft. **Xuan Dong:** Data curation. **Yilin Zhao:** Validation. **Sipan Chen:** Conceptualization, Writing – review & editing.

## Declaration of competing interest

The authors declare no conflicts of interest.
